# When politics meets policy: a realist review of how political context shapes the impact of public health legal interventions

**DOI:** 10.3389/fpubh.2025.1601467

**Published:** 2025-08-18

**Authors:** Yuri Lee, Jiwon Park

**Affiliations:** ^1^Department of Health and Medical Information, Myongji College, Seoul, Republic of Korea; ^2^Gyeonggi Public Health Policy Institute, Gyeonggi-do, Republic of Korea

**Keywords:** public health law, political determinants of health, realist review, context–mechanism–outcome (CMO) analysis, political context, legal epidemiology

## Abstract

**Background:**

Public health laws—whether focusing on taxation, bans, mandates, or licensing—are powerful tools for reducing risk behaviors and improving population health. However, identical legal interventions often produce starkly different outcomes across jurisdictions. Political and social contexts are increasingly recognized as key determinants of such variability.

**Objective:**

This study aimed to examine how and why public health legal interventions succeed or fail under different political circumstances, drawing on a Realist Review approach. We synthesized the interplay between legal epidemiology and political determinants of health to develop a deeper understanding of the mechanisms driving health policy outcomes.

**Methods:**

We followed RAMESES guidelines to identify and analyze 20 empirical studies, policy analyses, and global reports published from 2000 to 2023. We included sources that explicitly addressed both public health law or policy interventions and the political environment (e.g., trust in government, partisanship, lobbying, global donor influence). Using a Context–Mechanism–Outcome (CMO) framework, we coded and synthesized patterns to refine our initial program theory on how legal measures interact with political factors to shape health-related results.

**Results:**

Six recurring CMO patterns emerged. Laws are most effective when stable political leadership and public trust enable robust enforcement and funding. Conversely, fragmented governance or ideological polarization undermines or reverses legal interventions, especially those perceived as infringing personal freedoms (e.g., vaccine mandates, obesity restrictions). Industry lobbying frequently dilutes legislation, while external donor–driven policies can falter without sustained domestic support. Evolving moral and cultural attitudes likewise propel or hinder laws over time. We integrate these findings in a conceptual model demonstrating how political determinants modulate legal mechanisms, ultimately affecting population health outcomes.

**Conclusion:**

This Realist Review underscores that legal interventions alone cannot guarantee public health improvements. Rather, their success relies on supportive political contexts, coherent enforcement strategies, and alignment with evolving social values. Policymakers and advocates should anticipate and address political barriers—from partisanship to lobbying to donor dependency—to design and implement resilient, evidence-based public health laws. Future research should refine these insights using mixed-methods case studies and longitudinal evaluations, ensuring policy adaptations that optimize health equity and policy sustainability.

## 1 Introduction

Public health law has long been recognized as a powerful instrument for shaping health behaviors, reducing disease risks, and improving population-level outcomes (1). From tobacco taxes and smoke-free ordinances to mandatory vaccination regulations and nutrition labeling requirements, legal interventions are frequently employed by governments to address pressing health concerns (2, 3). These laws can influence not only individual behaviors—such as smoking cessation or vaccine uptake—but also the broader social and environmental conditions that support healthier lifestyles (4). Yet despite a growing array of evidence illustrating the potential efficacy of public health law, identical legal measures often yield widely disparate results across different jurisdictions and over time (5, 6).

A key factor behind these inconsistencies is the political environment in which laws are proposed, passed, and enforced (3). In many settings, political fragmentation, frequent leadership changes, or ideological polarization can diminish the capacity for robust lawmaking and implementation. For instance, tobacco taxes may be passed at the national level but remain ineffectively enforced at regional or local levels if relevant agencies are underfunded or face pressure from powerful interest groups (6). Similarly, vaccine mandates may reduce preventable diseases when they align with societal norms and enjoy strong political backing, but lead to backlash if partisan divides or distrust in government overshadow the public health rationale (5, 7).

To understand this variability, we must first clarify the core concepts that underpin this study. Public health law refers to the legal powers and duties of the state to ensure the conditions for people to be healthy, including legislation related to disease control, health promotion, and safety regulations (1). We define “political intervention” as the influence of political institutions, interest groups, and governance processes that either facilitate or obstruct the adoption, enforcement, and sustainability of public health law. For the purposes of this review, “political determinants” refer to institutional and sociopolitical conditions—such as governance structure, party dynamics, lobbying influence, and public trust—that shape the feasibility, effectiveness, and public reception of legal interventions (8, 9). These determinants operate within multi-level governance systems—local, national, and supranational (e.g., EU)—where separation of powers among executive, legislative, and judicial branches shapes political traction and legal implementation (10).

Although the importance of political context has been increasingly recognized in both academic and policy discussions, there is still a relative dearth of integrated frameworks explaining precisely how political factors interact with legal measures to yield specific health outcomes. Traditional systematic reviews frequently ask whether a certain law “works,” focusing primarily on effectiveness measures (1). However, such approaches can overlook the underlying mechanisms—like coalition-building, stakeholder engagement, and administrative capacity—through which political forces act to either bolster or undermine a policy’s impact. Scholars have thus called for more nuanced, theory-driven syntheses that examine the complex relationships among law, politics, and health (9).

In response to this need, the present study employs a Realist Review methodology, which seeks not merely to assess whether laws achieve their intended objectives, but to reveal the mechanisms by which they do (or do not) within varying contexts (11). By centering our analysis on the Context–Mechanism–Outcome (CMO) framework, we place equal emphasis on the political and administrative conditions that frame legal interventions, the mediating or moderating mechanisms that explain how such interventions unfold, and the ultimate health or policy results. This approach is particularly relevant for public health law research because of the diversity of legal instruments, the range of political settings in which they are applied, and the multi-level governance structures that can either facilitate or stifle policy implementation (1, 2).

The objectives of this review are twofold. First, we aim to identify and synthesize empirical and theoretical literature on public health law interventions that have examined political contexts, thereby highlighting how power dynamics, trust, lobbying, and other political variables influence outcomes across domains such as infectious disease control, chronic disease prevention, and environmental health. Second, we seek to refine an initial program theory that merges core elements of legal epidemiology (1) with political determinants of health (8), culminating in a conceptual model describing how and why certain laws achieve durable health improvements while others are weakened, reversed, or fail to gain traction.

## 2 Methods

### 2.1 Study design

We adopted a Realist Review approach to explore how and why public health legal interventions—such as taxation, bans, mandates, and licensing—succeed or fail under diverse political contexts (11, 12). Realist Review is particularly suited to analyzing complex social interventions because it moves beyond the question of “Does this intervention work?” to investigate “How, why, for whom, and under what circumstances does it work?” (13). This lens is compatible with our aim of synthesizing the interplay between legal epidemiology (1) and political determinants of health (8), recognizing that the effectiveness of laws depends substantially on the political environments in which they are enacted and enforced.

### 2.2 Research questions and initial program theory

We formulated two key research questions to guide the review. First, we asked which political conditions—including governance stability, party support, lobbying, and external aid—enable or hinder the implementation and effectiveness of public health legal interventions. Second, we examined the mechanisms (e.g., resource allocation, enforcement processes, stakeholder engagement, public trust) triggered within these varying contexts that lead to either improved or diminished health outcomes. These questions were grounded in our Initial Program Theory (IPT), which combined Burris et al. (1) framework of public health law research with Dawes (8) concept of political determinants. We hypothesized that legal interventions produce stronger health outcomes when (1) governance structures are stable or supportive, (2) there is adequate enforcement capacity, and (3) stakeholder conflicts are effectively managed. This IPT guided both data collection and analysis, enabling us to identify, confirm, or refine various Context–Mechanism–Outcome (CMO) pathways.

### 2.3 Search strategy

We followed the RAMESES (Realist and Meta-narrative Evidence Synthesis: Evolving Standards) guidelines (12) in conjunction with *Frontiers in Public Health* review recommendations to ensure methodological transparency and rigor. Searches were conducted in PubMed, Web of Science, Scopus, HeinOnline, EMBASE, and ProQuest, covering the period from January 1, 2000, to December 31, 2023. This range was chosen to capture contemporary public health policies and the political contexts influencing them. While we focused primarily on English-language articles, relevant non-English sources were included when accessible, especially for case studies illuminating unique political settings.

Search terms combined keywords and Medical Subject Headings (MeSH) related to legal interventions (“legal intervention,” “public health law,” “policy enforcement,” “tax,” “ban,” “mandate”), political determinants (“political context,” “political feasibility,” “governance,” “lobby,” “trust,” “partisanship”), and health outcomes (“health impact,” “health outcome,” “implementation,” “compliance”). Additional topic-specific terms (e.g., “vaccine mandate,” “tobacco control,” “soda tax,” “alcohol restriction,” “air pollution law”) were employed to ensure comprehensive retrieval. We also reviewed reference lists of key articles in a snowballing process, sought expert recommendations from colleagues in public health and political science, and included select gray literature (e.g., policy briefs, governmental reports) that offered substantive insights into political or legal contexts.

### 2.4 Study selection criteria and process

An initial pool of 217 articles was retrieved from database searches. After screening titles and abstracts for relevance and applying inclusion/exclusion criteria, 54 articles were assessed in full text. Of these, 20 studies were ultimately included based on their empirical focus on public health legal interventions and explicit discussion of political context. The selected studies represent a variety of national and subnational settings, with the majority originating from the United States, Australia, and Western Europe, and a few from lower- and middle-income countries.

We included studies if they (1) focused on a public health law or policy intervention (e.g., taxation, bans, mandatory programs), (2) explicitly discussed or analyzed political factors (e.g., government trust, partisan dynamics, lobbying, external donor influence), and (3) reported health-related outcomes or policy implementation outcomes (e.g., prevalence changes, mortality, compliance, sustainability).

We excluded clinical or biomedical studies without discussion of law, policy, or political context, as well as editorials or letters lacking methodological details or mentioning minimal political/legal dynamics. Studies with inaccessible full texts, which prevented thorough CMO analysis, were also excluded.

Titles and abstracts were initially screened for eligibility by a single reviewer. Articles deemed potentially relevant at this stage advanced to full-text review, where the same reviewer again applied the inclusion and exclusion criteria. In instances where the reviewer encountered uncertainty or ambiguity, a second reviewer was consulted, and any disagreements were resolved through discussion.

### 2.5 Data extraction and quality assessment

We designed a standardized charting form to capture key details from each study: study characteristics (authors, publication year, geographical setting, design), legal intervention (type, scope, domain), political factors (trust, lobbying, partisanship, donor influence), mechanisms (enforcement strategies, funding, stakeholder conflicts), and outcomes (health indicators, policy adoption, sustainability, unintended consequences). In line with Realist Review principles, we did not employ a numeric scoring system; rather, we appraised studies based on relevance (whether they enriched our understanding of the CMO linkages) and rigor (methodological clarity, data reliability, conceptual depth) (12). Studies deemed too methodologically weak or lacking sufficient context to inform our IPT were excluded.

### 2.6 Data analysis: realist synthesis

Analysis involved iteratively comparing extracted data against the Initial Program Theory. We coded each article’s content in terms of Context (political environments), Mechanisms (resource flows, enforcement processes, conflict/cooperation), and Outcomes (changes in health or policy indicators). Similar CMO dynamics were grouped into distinct patterns (e.g., “High trust + robust funding → consistent enforcement → improved health metrics,” “Polarization → public resistance → partial or ineffective law”). We then synthesized these patterns into broader themes, noting where they supported or contradicted our IPT. Finally, we produced a refined conceptual model illustrating how political contexts and legal interventions interact to drive or impede population health outcomes.

### 2.7 Ethical considerations

Because the study draws solely on previously published research and publicly available materials (e.g., government documents, academic databases), no direct data collection from human subjects occurred. Consequently, institutional review board (IRB) approval was not required. All sources were cited accurately and interpreted within the bounds of their respective publication processes, thereby upholding ethical standards regarding intellectual property and data reporting.

## 3 Limitations

Despite its potential to yield nuanced insights, this Realist Review faces certain constraints. First, the heterogeneity of included studies—ranging from quantitative surveys to qualitative case studies—limited direct comparison, particularly when political variables were not consistently reported. Second, focusing largely on English-language publications may have excluded pertinent literature from other languages, potentially restricting the range of political and cultural contexts examined. Finally, Realist Reviews emphasize explanatory depth over exhaustive breadth, which means that some relevant references may have been omitted if they did not provide enough detail on the political or legal environment. Nonetheless, by integrating a wide variety of sources and systematically examining the interplay between legal interventions and political contexts, this review offers an in-depth, context-rich synthesis that can inform both practice and research on public health law implementation.

Furthermore, the inclusion of studies spanning diverse policy domains—such as tobacco control, vaccination mandates, and environmental regulations—introduces challenges in terms of comparability. While this diversity enhances generalizability, it may limit the precision with which domain-specific causal mechanisms can be inferred. We attempted to mitigate this by focusing on common CMO patterns across domains yet acknowledge that some contextual nuances may be diluted in the synthesis.

## 4 Results

A total of 20 studies were ultimately included in this Realist Review, spanning a wide array of public health issues, legal interventions, and political contexts (see Table 1 for a descriptive summary of each reference). The public health domains most frequently addressed were tobacco control [e.g., (6, 14)], obesity and nutrition (3, 15), alcohol harm reduction (16, 17), infectious disease prevention via vaccine mandates (5, 7), and environmental health focusing on air pollution and related legislative measures (18). Although the largest proportion of included studies derived from the United States, Australia, and Western Europe, several comparative or global analyses (2, 6, 9) covered multiple countries or regions, including lower- and middle-income countries. The studies also spanned different levels of governance, including local ordinances, national legislation, and supranational frameworks such as EU directives and WHO conventions.

**Table 1:** Descriptive overview of included studies.

Study	Domain	Legal intervention	Key political factor	Study design	Key findings/insights
Attwell and Navin (5) Milbank Q	Vaccine mandates	Childhood vaccination mandates (scope, sanctions, severity)	Politicization of vaccine requirements; public skepticism or acceptance	Policy analysis (multiple jurisdictions)	Effective if mandates align with political norms and robust enforcement; strong resistance emerges where mandates clash with ideological beliefs.
Barry et al. (23) Psychiatric Services	Drug addiction and mental illness	Anti-discrimination laws, treatment policies	Public stigma, political readiness to fund mental health services	National survey (US)	Political and societal stigma can block effective laws or reduce funding, hindering improvements in health outcomes for marginalized groups.
Brownson et al. (2) Annu Rev. Public Health	Chronic disease prevention	Environmental and policy approaches (taxes, zoning, labeling)	Partisan differences in public health vs. individual responsibility	Narrative literature review	Policy-based interventions can be highly effective; lack of political consensus often stalls or dilutes chronic disease prevention measures.
Burris et al. (1) Milbank Q	Public health law systems and services	Intersection of public health law research (PHLR) and public health systems research	Varies by jurisdiction; synergy or fragmentation between legal and administrative frameworks	Conceptual discussion + case illustrations	Integration of legal research into broader public health systems is essential; political fragmentation reduces synergy and overall effectiveness.
Burris et al. (24) Public Health Reports	General public health law	5 essential public health law services (policy dev, enforcement, etc.)	Differential political support, resource allocation for law enforcement	Policy framework proposal	Outlines “essential services” model; underscores that local politics and funding streams shape success in implementing public health laws.
Elder et al. (16) Am J Prev Med	Alcohol harm reduction	Tax policy interventions for alcohol (excise taxes)	Varied state-level political acceptance of “sin taxes”	Systematic review of tax policy studies	Alcohol tax increases consistently reduce consumption; however, states with strong anti-tax sentiment implement weaker policies, limiting effect.
Gollust and Lynch (19) J Health Polit Policy Law	Healthcare attitudes and policy	Expanded coverage policies, social welfare interventions	Public attitudes toward deservingness of healthcare; political ideology	Survey-based study	Demonstrates how beliefs about personal responsibility intersect with politics, influencing public support for coverage-expanding laws.
Gostin (9) Global Health Law (book)	Global health governance	International legal frameworks (IHR, FCTC)	National sovereignty vs. global cooperation	Legal and policy analysis (global scope)	Success of global health laws depends on member states’ political will. National sovereignty claims can limit enforcement, impeding global cooperation.
Gravely et al. (6) The Lancet Public Health	Tobacco control	WHO FCTC demand-reduction measures (tax, packaging, advertising ban)	Multinational political structures; variable commitment to treaties	Cross-national association study (126 countries)	Countries with stronger political commitment and enforcement of FCTC measures see greater smoking prevalence declines, underscoring the importance of stable or supportive political environment.
Heikkila and Gerlak (25) Policy Studies J	Public policy learning	Collective learning approach in governance contexts	Emphasizes collaboration, stakeholder engagement, political culture	Conceptual approach + policy examples	Suggests that robust stakeholder collaboration is crucial when implementing laws; polarized or fragmented contexts hamper learning and diminish policy effectiveness.
Huang et al. (4) Prev Chronic Dis	Obesity policy	Systems-oriented, multi-level interventions (tax, labeling, environment changes)	Inter-sectoral collaboration requires political support and cross-agency coordination	Framework development + examples	Argues that obesity solutions need integrated approaches; political championing essential to sustain multi-level interventions.
Iarc Working Group on the Effectiveness of Tax and Price Policies for Tobacco Control (14) IARC Handbooks of Cancer Prevention	Tobacco control	Effectiveness of tax and price policies (FCTC)	Varying national legislative priorities and readiness	Meta-analysis + systematic review	Finds strong evidence that higher taxes reduce smoking, but political barriers often inhibit adopting sufficiently high rates or broad coverage.
Kypri et al. (17) Addiction	Alcohol harm reduction	Restricting pub closing times	Local political structure in Australian city; collaboration with police and councils	Before–after observational study (night-time assaults)	Found a significant reduction in assaults; local council support and stable leadership facilitated consistent enforcement.
Leichter (22) Milbank Q	History of “evil habits” vs. personal choices	Public attitudes and laws around tobacco, alcohol, diet	Long-term political shifts in moral, cultural perceptions	Historical/policy review	Over time, laws reflect changing public moral attitudes. Political readiness shapes how vigorously “harmful” behaviors are regulated.
Mello et al. (3) NEJM	Obesity and public health law	Menu labeling, marketing restrictions, litigation strategies	Strong corporate influence in policy debates; ideological framing of obesity as personal responsibility	Legal/policy commentary	Industry pressure often dilutes or delays obesity-related regulations. Political appetite for paternalistic interventions is limited, affecting law enactment and enforcement.
Parmet (20) Populations, Public Health, and the Law (book)	Population health law	Legal frameworks for balancing individual rights vs. population health	Varying judicial and legislative stances on “nanny state” critiques	Legal doctrine analysis	Courts and legislatures differ in how they weigh personal freedom vs. collective welfare; strong political or legal pushback can stifle protective laws.
Pomeranz et al. (21) Am J Public Health	Nutrition and obesity	Federal junk food and sugar-sweetened beverage tax feasibility	US legislative process, industry lobbying, partisan views on taxation	Legal and administrative feasibility analysis	Concludes that while legally possible, a national junk food tax faces significant political barriers (industry influence, anti-tax ideology) that weaken the chance of passage.
Reiter et al. (7) Vaccine	COVID-19 vaccination	Anticipated vaccine availability/mandates	US political polarization, distrust in federal government	National survey (U. S. adults)	Vaccine acceptability divided along partisan lines; potential enforcement challenges if mandates are introduced in polarized contexts.
Silver et al. (15) PLOS Med	Nutrition and obesity	Soda tax (Berkeley, California)	Strong grassroots advocacy, local referendum, politically aligned leadership	Before–after study (prices, sales, consumption)	Demonstrates meaningful reductions in sugar-sweetened beverage purchases; local political cohesion was critical to passing and implementing the tax effectively.
Zhang et al. (18) Nature	Environmental health (air pollution)	Legal limits on emissions + policy push for clean energy	Centralized Chinese govt approach; regional compliance variability	Policy commentary + secondary data on air quality	Rapid adoption of new standards at national level, but enforcement varied regionally; local political and economic interests sometimes overrode central directives.

In terms of legal or policy interventions, a considerable number of studies evaluated fiscal measures (such as excise taxes on tobacco, sugar-sweetened beverages, or alcohol), advertising and marketing restrictions (particularly for unhealthy foods and tobacco products), labeling requirements (menu labeling, warning labels), and mandated programs (vaccine requirements, smoke-free spaces). Several studies also described zoning and licensing strategies for restricting alcohol or tobacco retail outlets and environmental regulations (emissions caps, clean energy subsidies). Across these domains, the political context—whether stable or turbulent—consistently emerged as a key factor that shaped legal effectiveness.

Regarding methodologies, roughly half the included papers employed quantitative approaches—ranging from national surveys assessing public attitudes (7, 19) to cross-national association studies comparing policy strength and health outcomes (6). Others adopted qualitative case studies (17) or mixed-methods frameworks (15) that combined document analyses, stakeholder interviews, and empirical outcome data. A notable subset (2, 3, 20) offered policy commentaries or legal-analytic perspectives, elucidating how legislative processes or judicial interpretations shape the reach and durability of public health laws. Despite methodological variation, these studies consistently demonstrated that political determinants—such as lobbying, partisanship, government stability, and evolving cultural norms—play a decisive role in law enforcement and outcome sustainability.

A systematic review of each article’s Context (C), Mechanism (M), and Outcome (O) elements yielded six recurrent patterns, detailed in Table 2. While each pattern captures a distinct way that political contexts shape the mechanisms of lawmaking and enforcement, there is also considerable overlap. Below, we provide an expanded discussion of these patterns.

**Table 2:** Legal amendments, policy focus and programs, and ethical considerations on mental health system.

Pattern	Context (C)	Mechanism (M)	Outcome (O)	References	Interpretation/notes
Pattern 1	High Political Alignment and Trust - Unified leadership or stable coalition - Public trust in government	Strong Enforcement and Adequate Funding - Clear legal mandates + robust resource allocation - Effective public messaging	Tangible Health Improvements - Notable reductions in targeted risk factors (e.g., obesity, tobacco use) - Sustained public support and acceptance	Silver et al. (15), Kypri et al. (17), Gravely et al. (6), Brownson et al. (2), Burris et al. (24)	Unified political support leads to consistent implementation (e.g., soda tax, bar restrictions, FCTC measures) and fosters compliance. Public trust amplifies law effectiveness, resulting in measurable improvements in health outcomes.
Pattern 2	Fragmented/Unstable Governance - Frequent leadership changes - Low bureaucratic capacity	Weak or Inconsistent Implementation - Limited enforcement continuity - Conflicting policy signals	Minimal or Short-lived Health Gains - Laws enacted but not systematically enforced - Public confusion and low stakeholder engagement	Brownson et al. (2), Burris et al. (1)	Even if laws exist, political instability disrupts funding and enforcement, undermining long-term impact. External donors can help briefly but without local ownership or stable governance, benefits erode.
Pattern 3	Politicized/Polarized Issue - High media coverage, strong ideological divides	Public Contestation and Resistance - Frames of “nanny state” or overreach - Heightened lobbying/partisan conflict	Uneven Compliance or Policy Rollback - Significant pushback from opposition groups - Risk of mandates being weakened or repealed	(5), Reiter, Pennell and Katz (7), Mello et al. (3), Leichter (22)	Vaccine mandates, obesity rules, or public health ordinances become “hot button” issues. Political or ideological battles create enforcement gaps or lead to partial rollouts, limiting overall health gains.
Pattern 4	External/Global Influence - Donor-driven agendas or international treaties (IHR, FCTC)	Donor-Led or Treaty-Driven Enforcement - External funding, technical assistance - Domestic political buy-in varies	Variable Sustainability - Initial success if external impetus is strong - Potential policy collapse if donor priorities shift or local political ownership is weak	Gostin (9), Gravely et al. (6), Iarc Working Group on the Effectiveness of Tax and Price Policies for Tobacco Control (14)	Global frameworks or donor support can catalyze reforms but will not guarantee long-term success if local political leadership is ambivalent or opposes robust enforcement.
Pattern 5	Industry Resistance/Lobby Powe - Corporate influence in policy-making	Policy Capture or Dilution - Laws weakened during drafting or overshadowed in enforcement - Economic arguments trump public health	Limited Public Health Benefit - Laws enacted but with major loopholes - Lax enforcement or compromised scope	Mello et al. (3), Pomeranz et al. (21), Barry et al. (23)	Intense corporate lobbying (e.g., food, tobacco, alcohol industries) can water down legislation. Even if a policy passes, industry-driven exceptions, minimal enforcement, or ongoing legal challenges reduce overall impact.
Pattern 6	Shifts in Moral/Cultural Attitudes - Changing public norms over time	Evolving Legal Frameworks - Laws adjust to reflect emerging values (e.g., environmental concerns, addiction stigma, personal freedoms)	Dynamic Long-Term Outcomes - Laws can strengthen as norms shift (e.g., smoking bans) or weaken if moral panic fades	Leichter (22), Huang et al. (4), Parmet (20), Barry et al. (23)	Laws are neither static nor purely technical; they track moral/cultural changes. Political readiness to address new health threats (or revert to past norms) evolves, creating an ongoing cycle of reform, rollback, or reinvention.

Several studies (6, 15, 17) indicated that laws or policies aimed at reducing harmful consumption (e.g., tobacco use, excessive alcohol consumption, or sugary beverages) perform most effectively where there is cohesive political leadership and robust public trust in government. In these contexts, mechanisms such as stable funding flows, dedicated administrative support, and consistent policy messaging produce outcomes that include measurable declines in target behaviors and greater public acceptance. For instance, Kypri et al. (17) noted that restricting bar closing times in an Australian city was far more successful due to supportive local councils and well-funded policing efforts. Meanwhile, Silver et al. (15) found that Berkeley’s soda tax achieved a notable drop in sugar-sweetened beverage consumption precisely because city officials, health advocates, and the electorate were largely aligned in believing the tax would benefit public health.

In other settings, such as low- and middle-income countries undergoing political transition, frequent leadership turnover or limited bureaucratic capacity hindered the mechanisms needed for effective enforcement (1, 2). For example, transitions in donor-dependent governments often disrupted consistent follow-through of anti-smoking laws or environmental standards. As a result, laws may exist on paper but remain weakly enforced, yielding outcomes such as only modest or short-lived improvements (e.g., minor reductions in tobacco use, brief gains in air quality). This inconsistent enforcement also fosters public skepticism and lowers confidence in future policy efforts.

When an intervention directly touches on personal freedoms, ideological principles, or hot-button political disputes (e.g., vaccine mandates, obesity regulations), numerous authors (3, 5, 7) observed heightened public contestation. In such cases, the mechanism of conflict often overwhelms official enforcement channels, leading to partial or region-specific compliance. Reiter et al. (7), for example, documented that acceptability of COVID-19 vaccines in the United States varied widely by political ideology, suggesting that formal mandates—even if legislated—could spark significant backlash or non-compliance. Consequently, the outcome may be legal measures that fail to move the needle on vaccination uptake or are rolled back in politically conservative jurisdictions.

Several studies examined the interaction between international treaties or donor-led programs and domestic political will (6, 9). While global frameworks such as the WHO Framework Convention on Tobacco Control (FCTC) can jumpstart local legislation, the laws’ ongoing success depends on strong domestic ownership. Gravely et al. (6) found that countries more fully committed to FCTC guidelines (such as robust tobacco tax increases or plain packaging) saw greater declines in smoking prevalence.

A recurring theme (3, 19, 21) was the mechanism of industry-driven policy capture, whereby corporate actors in tobacco, alcohol, or food sectors exert significant influence over legislative drafting. This can result in weakened provisions—exemptions, watered-down language, delays in enforcement—ultimately producing outcomes in which resulting in policies that nominally exist but lack sufficient clarity or strength to drive meaningful behavior change. Mello et al. (3) concluded that, in the U. S. context, obesity-related laws (such as menu labeling or restrictions on high-calorie food marketing) often face formidable opposition from lobbying groups, preventing robust legislation from materializing.

Finally, Leichter (22), Barry et al. (23), and Parmet (20) showed that legal interventions do not operate in a cultural vacuum. Over time, evolving norms around personal responsibility, stigma toward certain behaviors (such as smoking or drug use), or environmental stewardship can reshape how laws are framed and enforced. Shifting cultural attitudes function as a mechanism that either fortifies laws (for instance, social endorsement of smoke-free spaces) or weakens them (if the public grows tired of paternalistic restrictions). These normative changes can produce long-term outcomes—like incremental expansions of regulatory frameworks—where successful legislation paves the way for stricter subsequent measures, or conversely, leads to backlash if moral support dissipates.

**Figure 1:**
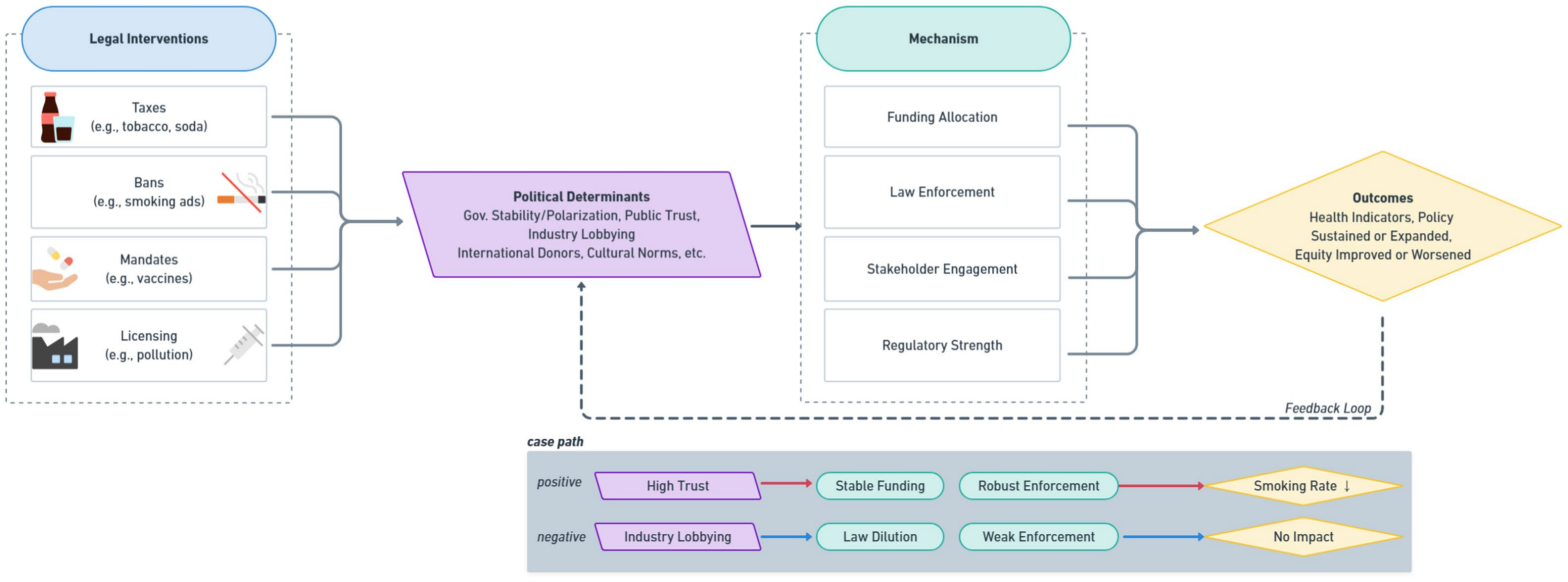
Integrated ‘legal-political determinants of health’ framework. This framework also illustrates contrasting case pathways. For instance, high trust may lead to stable funding and robust enforcement, resulting in reduced smoking rates. Conversely, industry lobbying may dilute legal provisions and weaken enforcement, resulting in limited or no health impact.

Figure 1 provides a consolidated framework illustrating how legal inputs interact with political determinants—such as trust, partisanship, industry influence, or cultural norms—to activate administrative mechanisms (e.g., funding, enforcement, public messaging), ultimately shaping health and policy outcomes. On the left side of Figure 1, various legal interventions—ranging from taxation and bans to licensing and mandates—are outlined as potential inputs. In the center, key political determinants—such as political trust, lobbying pressure, or cultural alignment—function as contextual filters that ultimately shape policy success or failure. On the right, the diagram designates health outcomes (e.g., changes in disease incidence, behavior patterns, or mortality) and policy outcomes (long-term durability, scope expansions or rollbacks).

Instead, they are profoundly shaped by the interplay of context (the political setting), mechanisms (enforcement practices, stakeholder mobilization, funding), and outcomes (health improvements or policy reversals). Cases of success—such as the Berkeley soda tax or rigorous enforcement of pub closing times in an Australian city—demonstrate how alignment among political actors, public trust, and clear legal authority can translate into tangible health gains. Meanwhile, less favorable contexts—marked by fragmented governance, social polarization, or vigorous corporate lobbying—frequently undermine or dilute well-intended laws. Moreover, some interventions show early promise under donor-driven or global frameworks but struggle to maintain momentum once external support diminishes, highlighting the importance of local political ownership.

Overall, this Realist Review confirms that lawmaking in public health is a dynamic, context-bound process, and that the same legal instrument may produce disparate outcomes across different jurisdictions or time periods due to variations in political alignment, resource availability, and social acceptance. By articulating these six key patterns and offering a conceptual synthesis (Figure 1), we provide both a diagnostic framework for understanding past or existing policy outcomes and a strategic lens for future interventions.

## 5 Discussion

Building upon the six patterns identified in the Results, this section interprets the mechanisms behind each pattern and connects them with existing theories and policy implications in the public health law literature.

The findings of this Realist Review highlight how political forces significantly mediate the impact of public health legal interventions, whether these interventions target tobacco use, obesity, infectious diseases, or environmental health hazards. By mapping diverse empirical studies and policy analyses onto a Context–Mechanism–Outcome (CMO) framework, we uncovered six main patterns that elucidate why identical legal measures can produce widely varying results across contexts. The following discussion interprets these results in light of previous research and explores the practical implications for policymakers, advocates, and scholars.

Our findings reaffirm that public health laws require supportive political conditions to ensure sustained impact. Our Pattern 1—high political alignment and trust—corresponds with evidence that governmental cohesion, stable leadership, and public support are critical factors enabling robust policy enforcement (15, 17). This aligns with prior work suggesting that a unifying political vision mobilizes resources for effective implementation (1, 2). Conversely, Pattern 2—fragmented or unstable governance—indicates that frequent turnover in leadership or under-resourced agencies can derail even well-crafted legislation. Thus, political stability can serve as both a facilitator and an outcome of well-functioning public health institutions, generating a reinforcing cycle in which strong governance structures bolster laws, which in turn maintain public trust.

Our review revealed that partisan polarization and industry lobbying often act as *countervailing mechanisms* to legislated health policies. As seen in Patterns 3 and 5, polarizing issues—such as vaccine mandates or food regulations—can become political flashpoints that hinder consistent enforcement and lead to partial or uneven compliance (5, 7). In parallel, powerful corporate interests (e.g., tobacco, alcohol, ultra-processed food industries) can significantly water down or delay policy enactment (3, 21). These findings resonate with broader critiques of “policy capture,” wherein well-resourced actors gain disproportionate influence, potentially overriding public health priorities (9). A major implication is that any strategy to strengthen legal interventions must incorporate political planning and capacity-building to limit the distortive effects of lobbying and partisan gridlock.

Pattern 4 underscored the role of international treaties (e.g., WHO Framework Convention on Tobacco Control) and donor-led initiatives (6). While such external impetus can galvanize domestic legislation—particularly in low- and middle-income countries—our review shows that *sustainability* depends on local political buy-in. This dynamic reflects the tension between global mandates and national sovereignty (9). Similar to the concept of “ownership” in development studies, the success of externally driven health laws hinges on whether political leaders integrate these measures into their own governance frameworks (2). Hence, capacity-building and stakeholder engagement within domestic institutions are essential if donor or treaty-based reforms are to endure beyond initial funding periods. The reviewed literature spans multiple governance levels, including local ordinances, national laws, and supranational frameworks such as EU directives or WHO conventions. These multi-level governance structures affect the feasibility and durability of legal interventions, as political determinants vary according to the scale and authority of implementation.

Our Pattern 6—shifts in moral or cultural norms—illustrates that public health laws are embedded in evolving social contexts (22, 23). Over time, behaviors once considered socially acceptable (e.g., indoor smoking, high sugar consumption) can become stigmatized, thereby *reinforcing* legislative measures (3). Conversely, public fatigue or changing cultural attitudes may weaken support for paternalistic regulations, leading to policy reversals or reduced compliance (20). This highlights the importance of long-term social marketing, public education, and community engagement that keep pace with shifting norms. Policymakers should view legal interventions not as static mandates but as *adaptive processes*, continually nurtured by public discourse and cultural alignment.

First, political feasibility assessments should precede or accompany any major public health law proposal. Mapping local governance structures, partisan fault lines, and stakeholder networks can clarify enforcement prospects and reveal potential allies or opponents (2). Second, multi-sectoral coalitions are pivotal for laws that provoke ideological contention. Engaging community groups, healthcare professionals, faith-based organizations, and business sectors might mitigate polarization, particularly when policy framings appeal to common values (19). Third, capacity-building within public agencies can minimize disruptions due to leadership turnover, ensuring that policy implementation is not derailed by political cycles (1). Fourth, international bodies and donors should prioritize local institutional strengthening rather than short-term compliance targets, thus fostering resilience if external funding or diplomatic focus shifts.

There are inherent limitations in any Realist Review, notably the dependence on secondary reporting of context, mechanisms, and outcomes, which may lack uniform detail across studies (11). Also, focusing predominantly on English-language literature means our synthesis may miss nuanced cases in non-English publications. Future research could involve prospective mixed-methods studies that integrate real-time political observations—such as legislative debates, lobbying disclosures, and media coverage—to trace law implementation more directly (10). Additionally, cross-national comparative analyses could further delineate how diverse governance regimes (e.g., federal, parliamentary, authoritarian) variably shape public health legal outcomes. Finally, exploring equity impacts of politically mediated laws—particularly among marginalized or disproportionately burdened populations—remains vital to ensuring that legal tools do not exacerbate health disparities (8).

These findings informed the development of an integrated conceptual model that illustrates how political determinants shape the implementation and effectiveness of legal public health interventions (Figure 1).

## 6 Conclusion

This review demonstrates that public health legal interventions operate within a dynamic political ecosystem, where governance structures, ideological climates, lobbying forces, cultural norms, and external influences collectively shape both short- and long-term outcomes. By adopting a Realist Review lens, we revealed *how* and *why* laws that appear promising on paper can falter in the face of political fragmentation, or conversely, succeed under conditions of stable leadership and strong public trust. Our integrative model clarifies that political determinants are not merely background “noise” but constitute core drivers that mediate law enforcement, resource allocation, and social acceptance (1, 8).

In practical terms, policymakers and advocates seeking to leverage the power of public health law should incorporate systematic political feasibility assessments and stakeholder engagement strategies from the earliest stages of law formulation. Cultivating political will—through intersectoral alliances, transparent communication, and robust administrative capacity—emerges as a key determinant of legal sustainability. Going forward, researchers can enrich this field by gathering direct, time-sensitive data on political processes, employing designs that capture the interplay between political shifts and legal adaptation. Ultimately, recognizing the reality that law is political is not just a theoretical insight but a practical imperative. This perspective enables more targeted, context-aware efforts to craft, implement, and maintain effective public health legislation over time. This review’s conceptual synthesis (see Figure 1) reinforces how these context-aware efforts must address the dynamic interplay between legal design, political feasibility, and administrative capacity. Unlike other domains of public regulation, public health law uniquely balances individual rights with collective risk management. What distinguishes public health law is its preventive focus, its reliance on scientific justification, and its demand for rapid yet democratically legitimate enforcement—features that are not always present in other domains of law.
